# Acquired Chiari I Malformation Secondary to Spontaneous Intracranial Hypotension Syndrome and Persistent Hypoglycemia: A Case Report

**DOI:** 10.4274/jcrpe.0042

**Published:** 2018-11-29

**Authors:** Hasan Önal, Atilla Ersen, Hakan Gemici, Erdal Adal, Serhat Güler, Serdar Sander, Sait Albayram

**Affiliations:** 1 University of Health Sciences, Kanuni Sultan Süleyman Training and Research Hospital, Clinic of Pediatric Endocrinology and Metabolism, İstanbul, Turkey; 2 University of Health Sciences, Okmeydanı Training and Research Hospital, Clinic of Pediatrics, İstanbul, Turkey; 3 University of Health Sciences, Kanuni Sultan Süleyman Training and Research Hospital, Clinic of Pediatrics, İstanbul, Turkey; 4 Bezmialem Vakıf University Faculty of Medicine, Department of Pediatric Neurology, İstanbul, Turkey; 5 University of Health Sciences, Kanuni Sultan Süleyman Training and Research Hospital, Clinic of Pediatric Surgery, İstanbul, Turkey; 6 İstanbul University Cerrahpaşa Faculty of Medicine, Department of Radiology, İstanbul, Turkey

**Keywords:** Intracranial hypotension, hypoglycemia, vagotomy

## Abstract

Spontaneous intracranial hypotension (SIH) is a rare and potentially serious condition in childhood. Cerebrospinal fluid (CSF) volume depletion is thought to be the main causative feature for intracranial hypotension and results from a spontaneous CSF leak, often at the spine level. SIH is increasingly diagnosed in clinical practice, although it manifests a varied symptomatology. The downward displacement of the brain, sometimes mimicking a Chiari I malformation, has rarely been reported. We present a case of a SIH with Chiari I malformation accompanied by an unusual clinical presentation of persistent hypoglycemia.

**What is already known on this topic?**

Spontaneous intracranial hypotension (SIH) is a rare and potentially serious pathological syndrome in childhood. Concomitant presentation of Chiari I malformation with SIH has rarely been reported. Diagnostic criteria wide-ranging due to variable clinical manifestations. Myelography computerized tomography and epidural blood patch are reliable diagnostic and treatment modalities.

**What this study adds?**

Chiari I malformation may mimic spontaneous intracranial hypotension (SIH) and to provide ideal therapy requires recognition of SIH. Persistent hypoglycemia was an early central feature of our patient which is an unusual finding in SIH. Some possible causes of hypoglycemia in SIH are discussed and we present vagotomy as a new treatment modality.

## Introduction

Spontaneous intracranial hypotension (SIH) is a rare condition with an estimated prevalence of only one in 50.000 individuals (1). The clinical spectrum of SIH is quite variable and includes headache, neck stiffness, cranial nerve dysfunction, radicular arm pain and symptoms of diencephalic or hindbrain herniation (1,2). Intracranial hypotension is a well-recognized sequel of a spontaneous cerebrospinal fluid (CSF) leak, particularly in cases in which the leak involves the thoracic spine (3). The cause for these CSF leaks remains unclear, but authors have postulated minor trauma, weakness of the dural sac or a combination of both (4,5). More cases are being diagnosed due to advances in imaging, but the diagnosis is still challenging because of the number of atypical, unconfirmed and doubtful cases. The current diagnostic criteria have a wide spectrum due to very variable clinical manifestations. The diagnosis of SIH is mainly based on presence of an orthostatic headache together with at least one of the following: low CSF pressure, sustained improvement of symptoms after epidural blood patching, demonstration of an active spinal CSF leak and cranial magnetic resonance imaging (MRI) changes demonstrating intracranial hypotension (6). Myelography computerized tomography is the most reliable method for the accurate localization of the CSF leak (7). In most cases, epidural blood patch is the main treatment modality (8).

Chiari I malformation is defined radiographically as a simple displacement of the cerebellar tonsils 5 mm or greater below the foramen magnum and is distinguished from Chiari II and Chiari III malformations occurring with myelodysplasia and cervical encephalocele, respectively (9). Spontaneous CSF leakage with development of SIH and acquired Chiari I malformation due to lumbar spinal CSF diversion procedures have both been well described. However, concomitant presentation of both syndromes has rarely been reported. Not to be confused with idiopathic Chiari I malformation, ideal therapy requires recognition of the syndrome and treatment directed to the site of the spinal CSF leak (10).

This is a case of a 13-year-old girl with acquired Chiari I malformation secondary to SIH with a unique coexistence of persistent hypoglycemia.

## Case Report

**Figure 1:**
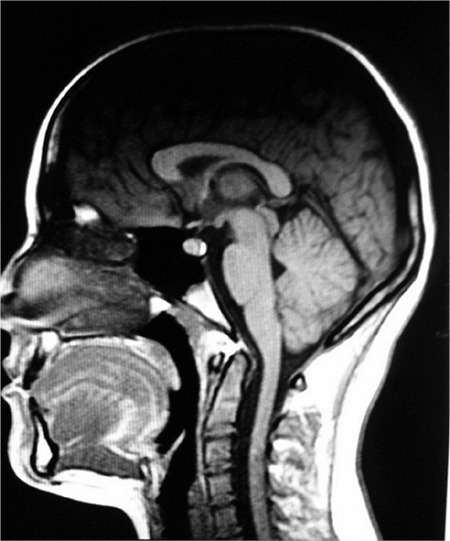
A simple displacement of the cerebellar tonsils 7 mm below the foramen magnum compatible with Chiari I malformation

A 13-year-old girl was admitted to the pediatric emergency unit of Kanuni Sultan Süleyman Training and Research Hospital suffering from hypoglycemia, syncope and convulsive seizures. She had no notable health problem in her past medical history up to 11.5 years of age. Thereafter, she had six subsequent hospital admissions in the previous 1.5 years, mostly at emergency services for hypoglycemic convulsions and syncope attacks. She was born at term, weighing 3100 g from non-consanguineous parents after an uncomplicated delivery. On evaluation of her records, it was found that she had undergone hypoglycemic periods 2-3 times per day, but syncope attacks were independent from hypoglycemic episodes. She was diagnosed with hyperinsulinemia with a serum glucose level of 29 mg/dL with a concomitant serum insulin level of 25 IU/L. Positron emission tomography and abdominal ultrasonography were performed to determine the etiology of hyperinsulinemia, but revealed normal anatomic findings. At neurological counseling electroencephalography showed bilateral delta waves with spikes and cranial MRI revealed a 7 mm herniation of the cerebellar tonsils from the foramen magnum (Figure 1). Further work-up with brainstem auditory evoked potentials and somatosensory evoked potentials, cardiac evaluation with echocardiography and holter monitoring revealed normal findings. Hypoglycemic episodes resolved in the following weeks but, although reduced in number, syncope episodes persisted. Pediatric psychiatry counseling results during her previous admission were not contributory.

**Figure 2:**
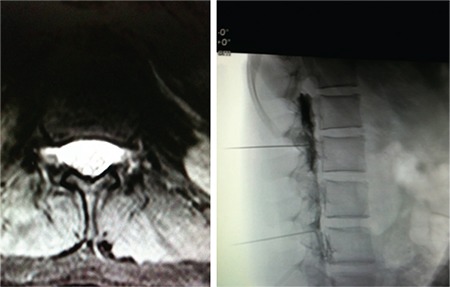
Two cerebrospinal fluid leaks at the lumbar 2 level and the procedure of autologous epidural blood patch

At her present admission, the patient’s body weight was 55 kg (90^th^ percentile) and height was 145 cm (25^th^ percentile). Laboratory investigations revealed hypoglycemia (serum glucose level: 30 mg/dL) with a high insulin level of 50 IU/L. Serum C-peptide level was 5 pmol/mL (N: 0.5-1.30 pmol/mL) and cortisol 23 µg/dL (N: 6.2-19.4 µg/dL). After intravenous glucose, intramuscular glucagon and methylprednisolone treatment, glucagon infusion was initiated. Glucose levels were 40 to 65 mg/dL during the first 24 hours, but surprisingly, glucose levels of 80-90 mg/dL were detected in the course of 24 hours after stopping the infusion. Oral glucose tolerance test (OGTT) showed hypoglycemia at the 30^th^ minute (glucose: 25 mg/dL) with an insulin level of 300 IU/L. We monitored our patient’s daily glucose levels by continuous glucose monitoring system [CGMS System Gold^®^ (Medtronic Minimed, Northridge, CA)] and 14 hypoglycemia episodes were noted, most occurring during sleep or defecation or in the postprandial period with a maximum glucose level of 70 mg/dL in three days of follow-up. We measured glucose levels before and after defecation; results were 85 mg/dL and 29 mg/dL respectively. No adrenergic symptoms were observed during hypoglycemic episodes. Diazoxide (40 mg/kg) and octreotide (40 µg/kg) treatment had no effect. Dysarthria was noted in the first month of hospitalization with frequent hypoglycemia episodes. During this period, syncope attacks were observed four times, independent of hypoglycemia. Additionally, the patient had severe biparietal headache episodes in the morning lasting for two hours. These were unrelated to hypoglycemia and were followed by anisocoria. Myelography was performed for SIH and verified with two CSF leaks originating at the lumbar 2 level. The patient underwent a procedure of autologous epidural blood patch at the CSF leak site (Figure 2), with good clinical results including complete control of her episodes of syncope, headache and hypoglycemia.

However, hypoglycemia recurred with dysarthria after two months and was attributed to displacement of the cerebellar tonsils, due to an epidural patch failure. Although the patient remains in good clinical condition after two subsequent epidural patch surgery interventions, neurologic problems and hypoglycemia persisted. Truncal vagotomy and partial pancreatectomy were planned for persistent hypoglycemia, because glucose levels were continuously under 29 mg/dL. At the initiation of this surgical intervention serum glucose was 25 mg/dL. Glucose level spontaneously normalized and was about 100 mg/dL during anesthesia. Truncal vagotomy was performed firstly. In order to test the efficiency of vagotomy, dextrose 10% solution infusion was initiated instead of 0.9% sodium chloride and serum glucose level increased to 180 mg/dL. Distal, partial pancreatectomy was performed additionally, to prevent another surgery risk. Post operatively diabetes mellitus developed. The patient was discharged with single-dose insulin glargine treatment and her follow-up has been successful for four years.

## Discussion

In clinical practice, SIH may manifest itself as a loss of the prepontine cistern due to leak of CSF, with flattening of the brainstem and downward herniation of the cerebellar tonsils, which may mistakenly lead to a diagnosis of Chiari I malformation (10,11). Our patient was diagnosed initially as a Chiari I malformation, but thereafter, with evaluation of symptoms and laboratory results, a diagnosis of SIH was reached.

SIH is initially suspected on the basis of presenting signs and symptoms such as headache, syncope and some neurological problems. However, in our patient, hypoglycemia was the leading clinical feature and this symptom is not a usual finding in SIH patients.

It has been suggested that hyperinsulinism is responsible for the hypoglycemia. Rekate et al (12) reported four cases diagnosed with Chiari malformation who were suffering from intermittent hyperinsulinemic hypoglycemia and proposed that vagal hypertonia, caused by variation in intracranial pressure, affected the pancreas leading to hypoglycemia in their patients. Tarani et al (13) proposed that the brainstem compression due to hindbrain herniation leads to dysfunction of the normal homeostatic mechanisms to correct hypoglycemia and direct stimulation of the vagal nuclei stimulates pancreatic islet cells to secrete insulin. In our patient, hypoglycemia mostly occurred during parasympathetic activities such as when in a postprandial state, defecation or sleep. Moreover, adrenergic activities had never been observed even with severe hypoglycemic episodes, thus this condition may be attributed to autonomic failure related to parasympathetic dominancy due to vagal stimulus. Vagal efferent activity starts with stimulus of oropharyngeal receptors by oral intake and increases with gastrointestinal peristaltic activity, consequently leading to insulin release, inhibition of norepinephrine from splanchnic nerves, gluconeogenesis and activation of glycogen synthesis (14). Vagal stimulus also produces an early phase of insulin response with postprandial insulin release (15,16). The occurrence of hypoglycemia at the 30^th^ minute of the OGTT with a high insulin level is noteworthy and supports a vagal effect. Moreover, in our opinion the syncope episode in our patient may be related to unbalanced reflexes of the sympathetic system (17).

SIH in childhood is rare (18). Occurrence of orthostatic headaches in the morning suggested a diagnosis of SIH and the myelogram showed two dural puncture areas in the lumbar region (Figure 2). Treatment with the patch procedure improved hypoglycemia and other symptoms with resolving caudal displacement of the cerebellar tonsils. Rekate et al (12) used continuous-drip feeding for hypoglycemia in their cases with Arnold Chiari syndrome. Unfortunately, persistent hypoglycemia relapsed after epidural patch procedure failures in our patient. In concordance with our estimation of parasympathetic dominancy, truncal vagotomy and partial pancreatectomy were planned as a radical therapy for her persistent hypoglycemia. Surgery was started with a serum glucose level of 25 mg/dL, and surprisingly, serum glucose level normalized with anesthesia induction. This outcome may be related to a possible effect of anesthesia on sympathetic-parasympathetic balance (19). Additionally, glucose response after the vagotomy procedure during surgery verified the role of a vagal effect on hypoglycemia. Diabetes mellitus presented after the surgery due to partial pancreatectomy and our patient continues to take single-dose glargine insulin treatment.

In clinical practice, intracranial hypotension syndrome may manifest with a variety of symptoms, one of which may be persistent hypoglycemia. This paper reports the findings in a patient with this syndrome, along with some pathophysiological considerations, diagnostic processes and possible treatment modalities in SIH patients with persistent hypoglycemia.
